# Cannabinoids, the Blood–Brain Barrier, and Neurodegeneration: Mechanisms, Dysregulation, and Therapeutic Perspectives

**DOI:** 10.3390/biom16020225

**Published:** 2026-02-02

**Authors:** Shimon Ben-Shabat, Ludmila Yarmolinsky, Nitzan Sharon, Taima Zeadnaa-Aldda, Shir Dayan, Boris Khalfin, Sigal Fleisher-Berkovich

**Affiliations:** Faculty of Health Sciences, Ben-Gurion University of the Negev, Beer-Sheva 8410501, Israel; yludmila@post.bgu.ac.il (L.Y.); kaunitz@post.bgu.ac.il (N.S.); taima@post.bgu.ac.il (T.Z.-A.); shirdayan09@gmail.com (S.D.); boriskh83@gmail.com (B.K.)

**Keywords:** cannabinoids, blood–brain barrier, neurodegeneration

## Abstract

Neurodegenerative diseases are a large and complex group of neurological disorders, including Alzheimer’s disease, Parkinson’s disease, Huntington’s disease, multiple sclerosis, and so on, which adversely affect the physical and mental health of millions of people globally. Unfortunately, these diseases currently have no cure; only symptomatic treatment is available. Therefore, there is still a growing interest in using cannabinoids to treat neurodegenerative diseases. This systematic review examines the interrelationship between cannabinoids, the blood–brain barrier, and neurodegeneration, and their mutual effects. The objective of this review is to provide an overview of the endocannabinoid system at the neurovascular interface, the alterations and dysregulation of the ECS in neurodegenerative diseases, the interactions of phytocannabinoids with the blood–brain barrier, and their therapeutic potential in the context of neurodegeneration. The findings may facilitate the targeted application of cannabinoids to address multiple aspects of neurodegenerative diseases.

## 1. Introduction

The comprehensive analysis of the influence of natural compounds on the proper functioning of living organisms and their use in disease treatment is one of the most important and advanced areas in medical sciences today. Particular attention is paid in these studies to cannabinoids. The classification of cannabinoids includes three large groups according to their origin: endocannabinoids, synthetic cannabinoids, and phytocannabinoids [1].

Endocannabinoids (or endogenous cannabinoids) are endogenous lipid signaling molecules that activate cannabinoid receptors [2].

**Figure 1:**
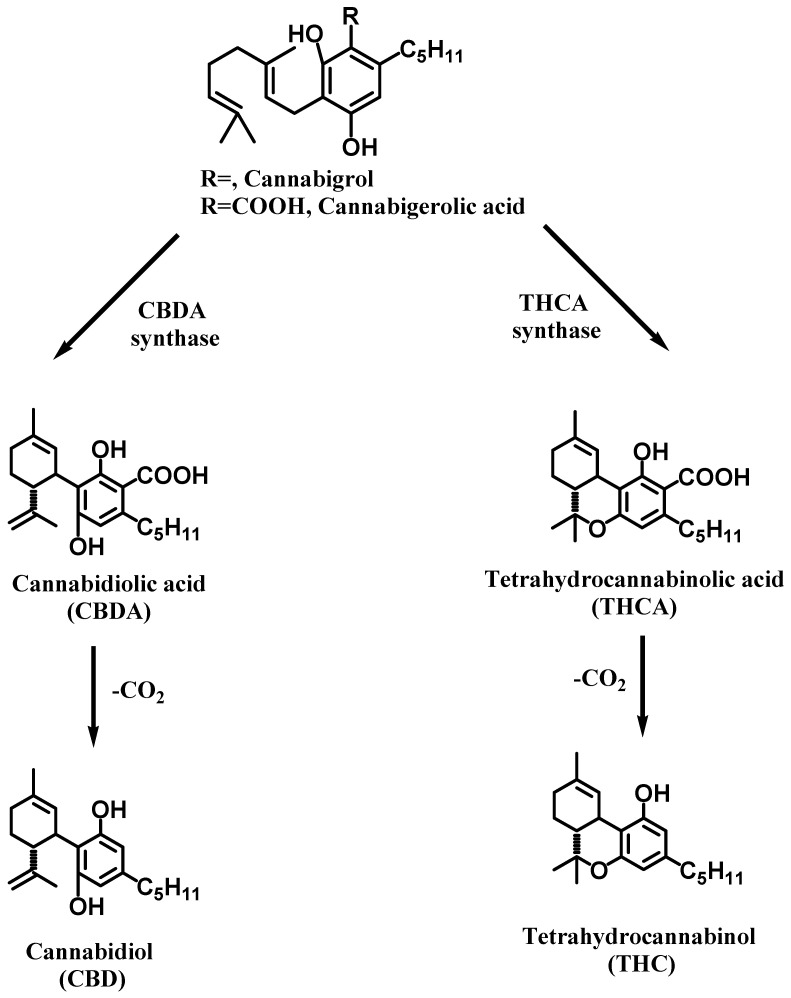
The molecular structure and the synthesis pathways of several cannabinoids.

Phytocannabinoids were first discovered in *Cannabis sativa* L. [3]; later, these compounds were also found in Rhododendron species, some legumes, the genus Radula, and some fungi [4]. From a chemical viewpoint, phytocannabinoids are meroterpenoids with a resorcinol core containing isoprenyl, alkyl, or aralkyl side chains (Figure 1) as a general rule [5]. More than 120 phytocannabinoids were identified in *Cannabis sativa* L. The parental molecule is cannabigerolic acid (CBGA) (Figure 1) from which many cannabinoids are synthesized; the dominant compounds tend to be Δ^9^-tetrahydrocannabinolic acid (Δ^9^-THCA) and cannabinolic acid (CBDA) [6].

More than 450 synthetic cannabinoids are known; they are structurally similar to phytocannabinoids and endocannabinoids [7].

Pharmacological studies have shown that cannabinoids possess anti-inflammatory, immunosuppressive, and antioxidant properties [8]. From the research and clinical perspectives, these properties are very important with regard to neurodegenerative diseases because the fundamental mechanisms underlying their development include aging, neuroinflammation, oxidative stress, mitochondrial dysfunction, apoptosis, protein disorders, and so on [9]. These diseases are a large and complex group of neurological disorders, including Alzheimer’s disease, Parkinson’s disease, Huntington’s disease, multiple sclerosis, amyotrophic lateral sclerosis, and so on, which adversely affect the physical and mental health of millions of people globally.

Although cannabinoids are promising agents for the treatment of neurodegenerative diseases [10,11], they may cause various short- and long-term side effects [12]. There is still a growing interest in the use of cannabinoids in this regard because neurodegenerative diseases have no cure at present, and only symptomatic treatment is available [8]. Progress in the development of cannabinoid-based drugs is impossible without a deep knowledge of the endocannabinoid system (ECS), cannabinoids, the blood–brain barrier (BBB), and neurodegeneration.

Therefore, understanding how cannabinoids, the blood–brain barrier, and neurodegeneration are interrelated and affect one another is vitally important for treating neurodegenerative diseases. Another important issue in this review is the endocannabinoid system. Its dysregulation plays a pivotal role in the pathophysiology of neurodegenerative diseases by facilitating neuroprotection and modulating the immune response. This review aims to provide an overview of the ECS at the neurovascular interface, alterations and dysregulation in the ECS during neurodegenerative diseases, phytocannabinoids interactions with the BBB, and their therapeutic potential in the context of neurodegeneration. The findings could inform the targeting of cannabinoids for various aspects of these multifactorial diseases.

## 2. Methods

We performed a structured literature search using several electronic databases (Science Direct, Google Scholar, PubMed, and Scopus). The search strategy was designed to determine the relationship between the ECS, BBB, and cannabinoids in the context of neurodegeneration on the basis of multiple criteria sorting methods [13]. The keywords were searched alone or in combination with Alzheimer’s disease, Parkinson’s disease, Huntington’s disease, and multiple sclerosis. The manuscripts published between 2000 and 2025 were chosen for possible inclusion in the present work. Narrative reviews, case reports or case series with 5 patients or fewer, letters, and editorials were excluded. A set of original research publications was obtained, and a comprehensive analysis of the interrelationship between cannabinoids, the blood–brain barrier and neurodegeneration were performed.

## 3. The Endocannabinoid System (ECS) and Blood–Brain Barrier Regulation

BBB forms a dynamic interface maintained by the neurovascular unit (NVU), which includes endothelial cells, pericytes, and glial cells (astrocytes and microglia) that together regulate vascular permeability, cerebral blood flow, and neuroimmune signaling [14]. The ECS is a critical modulatory network influencing sleep, immunity, reproduction, pain, memory, and BBB maintenance, and so on, comprising cannabinoid receptors, endogenous lipid ligands, and enzymes responsible for their synthesis and degradation [15].

Cannabinoid receptors CB1 and CB2 are G-protein-coupled receptors, with CB1 predominantly in the CNS mediating psychoactive effects, and CB2 mainly on immune cells, upregulated under pathological conditions [16]. Both receptors are also expressed on BBB cells, including endothelial cells, pericytes, and vascular cells [17,18]. They regulate the neurovascular interface by inhibiting leukocyte influx and adhesion molecules [19,20], maintaining tight junction proteins [21,22], and modulating neurotoxic mediators [15,23,24].

In stress-susceptible mice, the loss of the tight junction protein Claudin-5 in BBB endothelial cells triggers astrocytic CB1 upregulation, which helps preserve BBB integrity, indicating that ECS signaling contributes to stress resilience [22]. In drug-resistant mesial temporal lobe epilepsy, the human brain microvasculature exhibits reduced CB1/CB2 expression in the epileptogenic hippocampus and increased expression in the temporal neocortex, accompanied by enhanced Gαi/o coupling. These changes colocalize with tight junction proteins and correlate with BBB leakage, inflammation, and oxidative stress, highlighting CB1 and CB2 as key regulators of the neurovascular system [25]. However, CB1 activation in BBB endothelial cells can also induce oxidative stress and downregulate tight junction proteins, while astrocyte-specific CB1 knockout in EAE mice reduces BBB breakdown and vascular endothelial growth factor A-mediated permeability [17,26].

GPR55 is a third ECS receptor expressed in the brain and peripheral tissues. It is present in human brain microvascular endothelial cells, and its activation in rat endothelial cells transiently reduces barrier integrity and increases BBB permeability *in vivo* [27].

The main endocannabinoids, 2-arachidonoylglycerol (2-AG) and anandamide (AEA), are synthesized on demand from arachidonic acid-containing phospholipids via distinct enzymatic pathways, providing opportunities to modulate endocannabinoid signaling. AEA is synthesized primarily by NAPE-Phospholipase D (NAPE-PLD) and 2-AG by DAG Lipase (DAGLα/β). 2-AG functions as a full agonist at both CB1 and CB2 receptors, whereas AEA serves as a partial agonist primarily at CB1. In addition to CB1/CB2, AEA (and 2-AG to a lesser degree) activates other targets like TRPV1 (Transient Receptor Potential Vanilloid 1) and GPR55, expanding the scope of endocannabinoid signaling [28].

AEA and 2-AG are hydrolyzed to release free arachidonic acid by their degrading enzymes—fatty acid amide hydrolase (FAAH) and monoacylglycerol lipase (MAGL), respectively. These enzymes also degrade endocannabinoid-like amides, such as N-oleoyl-ethanolamine (OEA) and N-palmitoyl-ethanolamine (PEA), as well as oleamide. Oleamide is an agonist of CB1 receptors and peroxisome proliferator-activated receptors (PPARs) and is degraded by FAAH [29,30]. It has been found to inhibit gap junction communication in glial and microvascular endothelial cells and to increase barrier permeability in vitro [31,32]. OEA and PEA do not bind the classical cannabinoid receptors, and they activate receptors such as PPARα, which mediates their anti-inflammatory effects and protective effects on BBB integrity in vitro and *in vivo* [32,33,34]. PEA also acts via GPR55, and it can enhance the effects of AEA and 2-AG on CB1 and CB2 receptors and TRPV1 by preventing the breakdown of AEA and 2-AG, a mechanism known as the “entourage effect” [35,36,37].

ECS effects are context-dependent: under normal conditions, exogenous 2-AG or AEA can impair microvascular function by reducing endothelial proliferation, increasing apoptosis, and compromising vascular repair, whereas during pathological stress, they mitigate endothelial inflammation, increase the expression of tight junction molecules Claudin-5, ZO-1, and Beta-catenin, thus preserving BBB function [38,39,40].

Moreover, disrupted endocannabinoid metabolism (reduced levels of 2-AG and AEA with elevated degrading enzymes) has been associated with BBB impairment in rodent traumatic brain injury models [32,41,42,43].

The depletion of 2-AG, either by pharmacological or genetic blockade of DAGLα, compromises cultured brain endothelial monolayers (bEnd.3 cells), leading to fragmentation of VE-cadherin and downregulation of Z-1. In vivo, inhibition of DAGLα was associated with reduced 2-AG levels and decreased ZO-1 expression during BBB disruption [43,44].

MAGL is the primary enzyme controlling 2-AG hydrolysis in the brain. Pericytes are the primary source of MAGL in the nervous system, regulating the 2-AG/arachidonic acid ratio in a cell-specific manner. MAGL is important for maintaining BBB integrity. Inhibition of MAGL protects the BBB during inflammatory or ischemic injury through two main mechanisms: increasing 2-AG levels, which enhance protective cannabinoid signaling, and reducing arachidonic acid production, which drives inflammation. Part of this protective effect is mediated through CB1 and CB2 receptors, and the overall impact depends on the type of insult [45]. Recent work with a potent MAGL inhibitor (MAGLi 432) confirms its ability to modulate 2-AG and arachidonic acid in neurovascular cells but also highlights that inhibiting MAGL alone may not be sufficient to fully prevent BBB disruption in severe inflammatory conditions, suggesting that additional mechanisms contribute to BBB protection [46].

**Figure 2:**
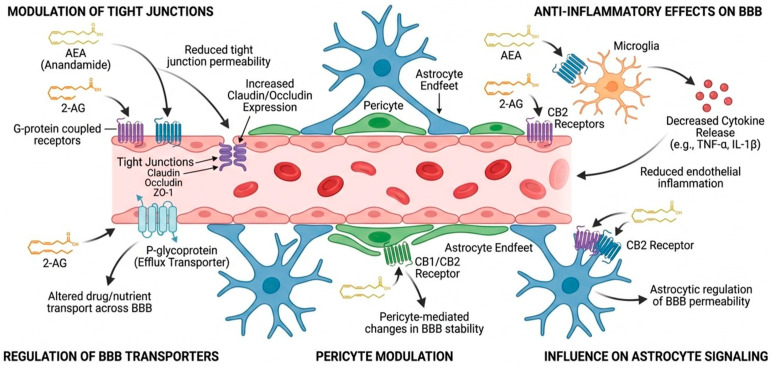
Effects of endogenous cannabinoids on the BBB.

Collectively, these findings underscore the importance of cellular context, receptor distribution, and inflammatory milieu in determining ECS effects at the neurovascular interface, highlighting the ECS as a complex yet promising target for neurovascular protection. Components of the endocannabinoid system at the neurovascular interface and their roles in blood–brain barrier regulation are summarized (Figure 2, Table 1).

## 4. Dysregulation of the Endocannabinoid System in Neurodegenerative Diseases

Mechanisms involving endocannabinoid levels, the upregulation and degradation of CB1 and CB2 receptors upregulation and degradation, and the main catabolic enzymes, namely two types of hydrolases—fatty acid amidohydrolases and monoacylglycerol lipase—play an important role in the pathophysiology of neurodegenerative diseases [51]. Various molecular processes (suppressing pro-inflammatory factors, promoting neuronal survival, shifting polarization of microglial cells, and so on) are modulated by CB1 [52]. CB2 may regulate inflammatory responses and maintain immune homeostasis [53].

It is known that the increased content of endocannabinoids is initiated by direct stimulation of the receptor by an endocannabinoid agonist or antagonist, and as a result, inhibitors of FAAH and MAGL raise the excitability of the ECS by decreasing the hydrolysis of endocannabinoids [54]. Although the comparable effects of FAAH, MAGL, and dual FAAH/MAGL inhibitors on neurodegeneration remain poorly understood, the majority of experimental studies on the basis of models of multiple sclerosis, Alzheimer’s, Parkinson’s, and Huntington’s diseases demonstrate their protective effect against neurodegenerative diseases [55,56]. Thus, FAAH and MAGL are promising therapeutic targets due to their roles in modulating endocannabinoid levels and in the regulation of neuroinflammation and neuroprotection.

**Figure 3:**
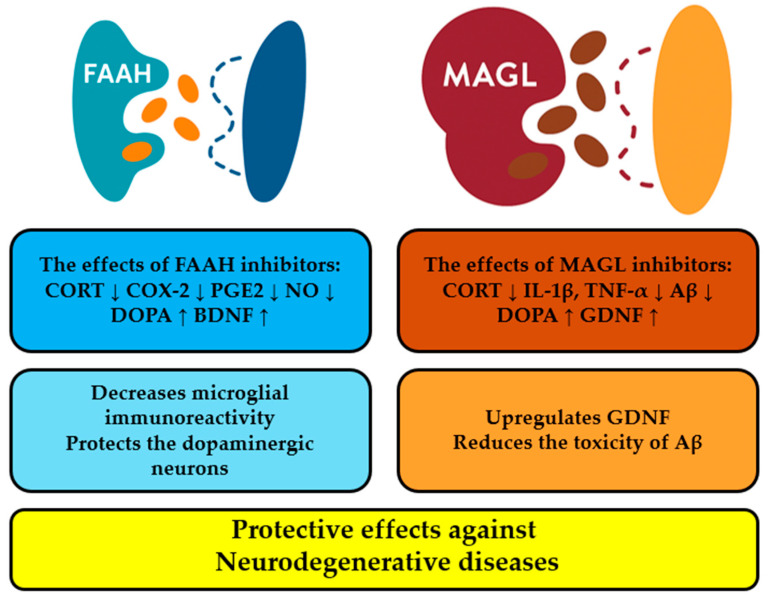
The effects of FAAH and MAGL inhibitors in animal models. The levels of the corticosterone (CORT), the cyclooxygenase (COX-2), prostaglandin E2 (PGE2) and nitric oxide (NO) decrease, while those of 3,4-dihydroxyphenylalanine (DOPA) and brain-derived neurotrophic factor (BDNF) increase (FAAH inhibition). MAGL inhibition leads to a significant decrease in the CORT level, cytokine expression, but DOPA levels and Glial cell line-derived neurotrophic factor (GDNF) expression increase. The down arrow means decrease of concentration; the up arrow shows its increase.

Figure 3 illustrates the interactions between hydrolase inhibition and various pathways. According to Figure 3, the effects of repeated FAAH inhibition have been investigated on the following biochemical indicators: the corticosterone (CORT) [57], the cyclooxygenase (COX) [58], prostaglandin E2 (PGE2) [59], nitric oxide (NO) [60], 3,4-dihydroxyphenylalanine (DOPA) [61] and brain-derived neurotrophic factor (BDNF) [62]. Figure 3 shows that short-term pharmacological inhibition of MAGL influences the CORT level [63], cytokine expression [64], DOPA response [65] and Glial cell line-derived neurotrophic factor (GDNF) expression [66].

In addition, GDNF is of decisive importance for the maintenance of the nigrostriatal system [67] and an upregulation of GDNF acts against neurotoxicity in Parkinson’s disease [68].

CB1 and CB2 modulations cause a decrease in microglial activation and inflammatory cytokines IL-2 and IL-6 in Alzheimer’s disease [69].

The inhibition of MAGL and FAAH is a potential possibility to suppress inflammation, prevent neurodegeneration, improve synaptic plasticity, and boost spatial learning [70]. The comparable effects of FAAH, MAGL, and dual FAAH/MAGL inhibitors on neurodegeneration remain poorly understood.

It is interesting that MAGL but not FAAH inactivation inhibits neuroinflammation and allows for avoidance of neurodegeneration in a mouse model of Parkinson’s disease [65]. MAGL inhibition promotes normalization of arachidonic acid (AA) and 2-AG dynamics in various models of multiple sclerosis, Parkinson’s disease, and Alzheimer’s disease [71]. Perhaps inhibition of MAGL adjusts prostaglandin production and diminishes inflammation in neurodegenerative diseases [72].

Overexpression of CB2 and FAAH enzymes has been found after postmortem analysis of brains from Alzheimer’s disease patients, and increased amyloid beta plaque formations have been recorded [73].

According to the *in vivo* study, the MAGL inhibitor is not able to change cognitive deficits associated with sporadic Alzheimer’s disease, but it improves some biochemical properties [74]. Systemic administration of MAGL/FAAH inhibitory compounds improves memory impairments in rodent models of Alzheimer’s disease [75].

Future studies should focus on the catalytic mechanisms and regulation of both hydrolases to develop effective inhibitors.

## 5. Phytocannabinoid Modulation of Blood–Brain Barrier Integrity

### 5.1. The Blood–Brain Barrier

The BBB is a highly selective semipermeable interface that separates the central nervous system from the peripheral circulation. As mentioned previously, it is formed primarily by brain microvascular endothelial cells joined by tight junction (TJ) proteins, and supported by the basement membrane, pericytes, and astrocyte end feet. Together with perivascular components such as microglia and neurons, this structure constitutes the NVU [76].

Functionally, the BBB maintains CNS homeostasis using physical, metabolic, and transport barriers. These barriers enable the strict regulation of paracellular diffusion, transcytosis, and solute flux through specific transport systems and metabolic enzymes, allowing the entry of essential nutrients while removing potentially harmful compounds, including most pathogens, toxins, and systemically administered drugs [77,78].

### 5.2. Phytocannabinoids and BBB Penetration

**Figure 4:**
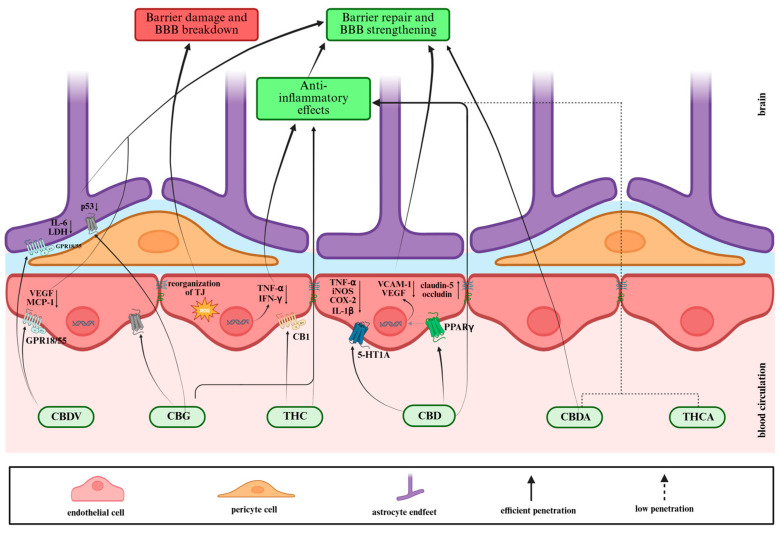
A schematic illustration of phytocannabinoids’ effects on the BBB.

Figure 4 shows phytocannabinoids’ effects on the BBB. Phytocannabinoids, as mentioned, are lipid-soluble, plant-derived cannabinoid compounds, which include major neutral molecules such as delta-9-tetrahydrocannabinol, cannabidiol (THC), cannabidiol (CBD), and cannabigerol (CBG), along with their acidic precursors like tetrahydrocannabinol acid (THCA), cannabidiol acid (CBDA), and cannabigerolic acid (CBGA). Their natural compounds’ high lipophilicity enables passive diffusion across the BBB, allowing them to interact with endothelial cells, TJs, and other components of the neurovascular unit, thereby influencing BBB permeability, neuroinflammation, and brain distribution [79,80].

The mechanisms underlying cannabinoid delivery to the brain remain incompletely defined, in part due to variability in the routes of administration used for cannabinoid delivery. Neutral cannabinoids such as THC and CBD are typically described as highly lipophilic, enabling them to cross the BBB more readily than their acidic counterparts (THCA and CBDA). Despite similar lipophilicity among neutral cannabinoids, their brain-to-plasma exposure differs due to structural features such as molecular rigidity and polar surface area, as well as formulation and route of administration [80,81].

### 5.3. Neutral Phytocannabinoids and the BBB

Beyond classical endocannabinoid system signaling, CBD and THC can modulate BBB function through several non-classical targets such as PPARγ, serotonergic receptors (5-HT1A), TRP channels, and redox-sensitive signaling cascades that influence endothelial survival, TJs, and inflammatory status [82].

In an in vitro human BBB model exposed to oxygen–glucose deprivation (OGD), CBD reduced barrier hyperpermeability and endothelial damage. This protective effect was blocked by PPARγ and 5-HT1A antagonists, while CB1/CB2 antagonism had no effect, indicating a cannabinoid-receptor-independent mechanism at the BBB [82]. CBD also altered the expression of factors such as vascular cell adhesion molecule-1 (VCAM-1) and vascular endothelial growth factor (VEGF) in brain endothelial cells via PPARγ activation [82]. This suggests that its vascular-protective actions are driven by transcriptional regulation of adhesion and angiogenic pathways, rather than by activation of classical cannabinoid receptors.

CBD was also reported to counteract the inflammatory activation and barrier disruption of endothelial cells exposed to high-glucose conditions [83]. Additionally, CBD enhances the transport of lipid nanocapsules across the BBB in both hCMEC/D3 in vitro permeability assays and mouse biodistribution studies, with the smallest cannabinoid-decorated nanocapsules showing the highest brain-targeting efficiency [84].

In an *in vivo* endotoxin shock model of encephalomyelitis, CBD effectively protected the BBB from the loss caused by intravenous administration of LPS. This protective effect is attributed to the downregulation of tumor necrosis factor-alpha (TNF-α), cyclooxygenase-2 (COX-2), and inducible nitric oxide synthase (iNOS), as confirmed by quantitative real-time PCR [85].

Furthermore, CBD was reported to decrease aquaporin-4 (AQP4)- and GFAP-positive cells, suppress pro-inflammatory cytokines (TNF-α and IL-1β), and upregulate the TJs proteins (claudin-5 and occludin) in the traumatic brain injury (TBI) model. CBD also reduced brain water content and BBB breakdown following TBI, leading to improved neurological status. Overall, CBD strengthened BBB integrity and diminished post-TBI edema [86].

Interestingly, THC exhibits dual effects: while its high lipophilicity allows rapid BBB penetration, it can also impair barrier integrity through oxidative stress, TJs reorganization, and cytoskeletal alterations in brain microvascular endothelial cells. This barrier damage results from redox imbalance and activation of stress-responsive signaling pathways. Although CB1 activation contributes to these effects, the downstream damage appears to be driven primarily by oxidative mechanisms and TJs remodeling [17].

At lower doses (e.g., 10 mg/kg), THC may indirectly enhance BBB stability through its anti-inflammatory effects. Several articles suggest that THC may contribute to reducing neuroinflammation and thereby indirectly enhance BBB integrity, particularly when combined with CBD [87,88,89]. However, direct protective actions of THC on BBB endothelial monolayers are less consistently demonstrated than those of CBD, which emphasizes the need to define dose, exposure time, and inflammatory context when evaluating THC’s overall impact on BBB function [80,90].

Recent findings show that CBG and cannabidivarin (CBDV) exert a protective effect on the three cell types of the triple-culture BBB in vitro OGD model. In astrocytes, both cannabinoid compounds decrease the release of interleukin-6 (IL-6) and lactate dehydrogenase (LDH), while CBDV additionally decreased the secretion of VEGF. CBDV also decreased MCP-1 in brain endothelial cells. CBG diminishes DNA-damage markers, whereas CBDV increases them. Their effects on astrocytic LDH were not altered by blockade of major cannabinoid, PPAR, serotonin, or TRP receptors; CBDV acted partly through GPR55 and GPR18, while no specific target was identified for CBG [79].

### 5.4. Acidic Phytocannabinoids and the BBB

Acidic cannabinoids such as THCA and CBDA remain significantly less investigated than their neutral counterparts (THC and CBD) in the context of BBB function. Current data is limited to pharmacokinetic profiling and indirect neuroprotective outcomes rather than direct effects on BBB components [81,91].

Despite their limited BBB penetration, THCA and CBDA cross the BBB in sufficient amounts to alleviate Aβ/tau pathology and neuroinflammation in Alzheimer’s models, demonstrating indirect preservation of barrier integrity. Preliminary data from triple-culture BBB in vitro models reveal that CBDA can alleviate ischemia-triggered increases in permeability, similar to CBD [92]. Nevertheless, more in-depth studies about the precise effects of acidic cannabinoids on TJs organization and expression, endothelial signaling, and barrier integrity are needed [91,93].

## 6. Neuroprotective and Therapeutic Roles of Phytocannabinoids

Neurodegenerative disorders, such as Alzheimer’s disease and Parkinson’s disease, share a prominent component of chronic neuroinflammation [94,95].

Microglial cells and astrocytes play key roles in regulating neuroinflammatory processes [96]. Glial cells also contribute to the formation and maintenance of the impermeable blood–brain barrier, which prevents potentially toxic blood-borne substances from entering the brain [97].

Cannabinoids can beneficially modulate these neuroimmune responses and may contribute to the preservation of BBB integrity under inflammatory conditions, thereby supporting their potential relevance in neuroprotective strategies rather than establishing direct therapeutic efficacy [23,98,99,100].

At this neurovascular interface, the endocannabinoid system becomes particularly relevant. Endogenous cannabinoids such as anandamide and 2-AG are synthesized locally by endothelial cells, astrocytes, and microglia, enabling rapid modulation of tight junction dynamics, vascular tone, and leukocyte trafficking [23,98,99,100].

In models of LPS-induced and ischemia-related inflammation, cannabinoid treatment preserves tight-junction structure and reduces BBB permeability. Furthermore, CBD or selective CB2 agonists decrease endothelial expression of ICAM-1 and VCAM-1, thereby limiting leukocyte adhesion and trans-endothelial migration, while increasing trans-endothelial electrical resistance, a functional marker of tight-junction reinforcement. These findings suggest that CBD may exert protective and beneficial effects in inflammatory diseases of the nervous system, including epilepsy, psychiatric disorders, and neurodegenerative conditions. These vascular effects involve not only receptor activation but also suppression of NF-κB-dependent transcriptional programs within endothelial cells. Additionally, cannabinoids modulate the RhoA/ROCK signaling pathway, a pathway that regulates cytoskeletal tension and tight junction stability, providing a mechanistic link between ECS activation and structural BBB preservation [80].

During inflammatory activation, microglial CB2 receptor expression increases, allowing cannabinoids to selectively modulate pro-inflammatory (M1-like) phenotypes while sparing or enhancing protective, phagocytic functions (M2-like). This selective immunomodulation distinguishes cannabinoids from broad immunosuppressants [101,102].

Accordingly, microglial activation is a regulatable process and has emerged as a therapeutic target in neurodegenerative diseases. Chronic and dysregulated microglial activation is a hallmark of several neurodegenerative disorders, including Alzheimer’s and Parkinson’s diseases, where sustained production of pro-inflammatory cytokines contributes to progressive neuronal damage. Nicotinic acetylcholine receptors (nAChRs) expressed in neurons, as well as microglia and astrocytes, participate in the cholinergic anti-inflammatory pathway activated by vagus nerve stimulation [103,104,105]. This pathway has been implicated in limiting neuroinflammation-driven neurodegeneration by suppressing innate immune activation within the CNS. Experimental studies show that stimulating these receptors attenuates LPS-induced cytokine production in macrophages and microglial cells [106,107,108]. For example, activation of the α7 nAChR by nicotine markedly reduces the ischemia-induced expression of TNF-α and IL-1β, indicating α7 nAChR-dependent suppression of microglial activation. Given the overlap between ischemia-induced inflammation and inflammatory mechanisms observed in chronic neurodegenerative diseases, modulation of α7 nAChR signaling may represent a potential strategy for attenuating microglia-mediated neurodegeneration [109].

Building on this concept, selective α7 nAChR agonists, such as GTS-21, demonstrate anti-inflammatory and neuroprotective effects in mouse models of neuroinflammation. In LPS-induced Parkinsonian inflammation and in MPTP-based PD models, GTS-21 reduces the expression of iNOS and pro-inflammatory cytokines. At the same time, it increases the levels of the anti-inflammatory mediator TGF-β. These effects coincide with the suppression of NF-κB signaling and the activation of PPARγ-dependent pathways. In MPTP-treated mice, GTS-21 alleviates motor deficits and reduces microglial activation, linking cholinergic modulation of microglia to structural and functional neuroprotection in dopaminergic degeneration [95].

Astrocytes maintain CNS homeostasis by regulating cerebral blood flow, supporting neuronal metabolism, and modulating neuronal function through cytokine secretion [97]. Under inflammatory conditions, astrocytes become reactive and produce molecules that inhibit axonal regeneration, while releasing cytokines and chemokines that shape CNS immune responses [110]. Astrocytes also prevent glutamate-mediated excitotoxicity by rapidly clearing synaptic glutamate, thus avoiding excessive glutamate receptor activation, pathological Ca^2+^ influx, and ionic imbalance [97,111]. In neurodegenerative diseases such as Alzheimer’s disease, Huntington’s disease, and amyotrophic lateral sclerosis, failure of this astrocyte-dependent glutamate buffering system promotes sustained excitotoxic stress and progressive neuronal loss [112].

Reactive astrocytes comprise functionally distinct subtypes. Pro-inflammatory A1 astrocytes are induced by microglia-derived cytokines and exhibit neurotoxic properties, impairing neuronal development and promoting the death of both neurons and oligodendrocytes, whereas A2 astrocytes support neuronal survival and repair mechanisms. Modulation of astrocytic phenotypes therefore represents a potential mechanism through which cannabinoids may influence neuroinflammatory environments in diseases such as Alzheimer’s disease, Huntington’s disease, Parkinson’s disease, multiple sclerosis, and amyotrophic lateral sclerosis [113]. CB1 has an important role in modulating stress responses *in vivo* models. Understanding the beneficial endocannabinoid-related adaptations within the BBB can represent a promising strategy for developing innovative therapies for neurodegenerative diseases [22].

Cannabinoids modulate excitotoxicity by regulating glutamate release through CB1 receptors on presynaptic terminals. CB1 signaling controls excessive neurotransmitter release partly by influencing voltage-gated calcium channels. Additionally, CB1-mediated inhibition of mitochondrial respiration in axonal terminals may reduce metabolic stress during inflammatory insults, thereby providing further protection against excitotoxic damage [114,115].

Neuroinflammation also induces excessive production of reactive oxygen and nitrogen species, creating oxidative stress that damages membrane lipids, proteins, nucleic acids, and mitochondria, ultimately promoting cell death. In multiple sclerosis (MS), cannabinoids may protect against oxidative injury by limiting nitric oxide production in macrophages/microglia and astrocytes [116,117,118,119].

Phytocannabinoids, particularly CBD, reduce the generation of reactive species while enhancing endogenous antioxidant defenses and mitochondrial resilience. CBD activates the Nrf2 pathway and upregulates downstream targets, such as heme oxygenase-1 (HO-1), thereby strengthening the cell’s antioxidant capacity. In the brain, Nrf2 further contributes to neuroprotection by mitigating neuroinflammation triggered by harmful stimuli commonly observed in neurodegenerative diseases. Through modulation of pathways such as the NLRP3 inflammasome, Nrf2 plays a central defensive role against oxidative stress, gliosis, protein aggregation, and inflammatory damage, thereby reinforcing the neuroprotective potential of CBD in disorders such as Alzheimer’s and Parkinson’s disease. By stabilizing mitochondrial membrane potential and reducing cytochrome c-dependent caspase activation, CBD further attenuates apoptosis under conditions of oxidative and neuroinflammatory stress, adding an additional mechanistic layer to its neuroprotective profile [120,121,122].

Accumulating evidence indicates that phytocannabinoids, such as CBG, Δ^9^-THC, and CBD, interact with multiple targets in the ECS and beyond. In addition to CB1 and CB2 receptors, they modulate TRP channels (TRPV, TRPA, and TRPM), GPR55, voltage-gated calcium and sodium channels, and transcriptional regulators, including NF-κB [123,124]. Through modulation of NF-κB and these ion channels, phytocannabinoids can attenuate neuroinflammatory signaling and reduce the expression of pro-inflammatory cytokines and chemokines, processes that are critically involved in the pathogenesis of neurodegenerative diseases such as Alzheimer’s and Parkinson’s disease. NF-κB serves as a key regulator of immune function by driving the expression of pro-inflammatory genes, including those encoding cytokines and chemokines, and by controlling the activation, differentiation, and effector functions of innate immune cells and inflammatory T cells [125].

Another important non-cannabinoid target is PPARγ, which represses pro-inflammatory gene expression and counteracts NF-κB activity. PPARα and PPARγ agonism is therefore of growing interest as a therapeutic mechanism through which phytocannabinoids may act in neuroinflammatory conditions [123,124,126,127,128]. In this context, CBD acts as a PPARγ agonist, attenuating NF-κB signaling. PPARγ enhances lipid accumulation in both mice and humans and is involved in the pro-apoptotic and tumor-regressive effects of CBD. CBD induces an increase in COX-2–dependent prostaglandin levels, promoting PPARγ translocation to the nucleus and consequently triggering apoptotic cell death. PPARγ represents a critical factor in CBD’s ability to modulate inflammatory responses [129,130].

Based on the role in modulating neuroinflammation and stabilizing the BBB, both preclinical and clinical studies have evaluated phytocannabinoids in neurodegenerative disorders, such as AD, PD, Huntington’s disease, ALS and MS. Studies show that Δ^9^-THC reduces pro-inflammatory cytokines, including IFN-γ and TNF-α, and suppresses T-cell proliferation, whereas CBD and CBG reduce inflammation and pain and improve motor function [131,132].

In AD models, studies demonstrated that various phytocannabinoids, including CBD, THC, and CBG, reduce neuroinflammation and protect against intracellular amyloid-β (Aβ) toxicity, promote the breakdown of aggregates, decrease the expression of TNF-α, COX-2, and IL-6, and contribute to improved cognitive performance. Moreover, CBD was found to reduce oxytosis, a cell death pathway associated with oxidative stress. Mechanistically, phytocannabinoids exert these neuroprotective effects not only through classical CB1 and CB2 receptor-mediated signaling but also by modulating oxidative stress responses, mitochondrial function, and proteostasis pathways. In particular, CBD and cannabigerol (CBG) have been shown to enhance cellular antioxidant defenses, reduce the accumulation of reactive oxygen species (ROS), and preserve mitochondrial bioenergetics in neurons exposed to Aβ-induced toxicity. Additionally, these compounds enhance protein clearance mechanisms, thereby mitigating proteotoxic stress and reducing the accumulation of misfolded Aβ aggregates, which may support neuronal survival and function. Collectively, these findings indicate that phytocannabinoids act through a multifaceted neuroprotective network, making them promising candidates for therapeutic intervention in Alzheimer’s disease [133,134,135,136]. In a recent study, CBG, CBDV, and acidic cannabinoids have been demonstrated to have neuroprotective properties in PC12 cells; these compounds inhibit amyloid β-evoked neurotoxicity [137]. Another computational and biochemical research investigating Alzheimer’s disease *in vivo* revealed that repeated treatment with CBDA and CBGA may prevent aggregation of β-amyloid fibrils and restore the expression level of TRPM7 according to the β-arrestin assay on GPR109A and qPCR [138].

In Parkinson’s disease models, CBD reduces dopaminergic degeneration, shifts microglial activity toward an anti-inflammatory state, and improves motor performance. Additionally, CBD increases TRPV1 expression in astrocytes, which may support repair processes [139].

In models of Huntington’s disease, Δ^9^-THC has been shown to attenuate motor coordination deficits and reduce both striatal degeneration and the accumulation of huntingtin protein aggregates in R6/2 transgenic mice. Cannabigerol (CBG) has demonstrated neuroprotective effects in multiple Huntington’s disease models, including a 3-nitropropionate-induced model and transgenic mice. In the 3-nitropropionate model, CBG significantly decreased neuronal death; lowered the levels of pro-inflammatory mediators, including COX-2, inducible nitric oxide, TNF-α, and IL-6; and improved motor performance. In transgenic mice, CBG treatment produced modest improvements in motor coordination, partially normalized genes dysregulated in Huntington’s disease, and enhanced the expression of anti-inflammatory mediators, including PPARγ [140,141,142].

In ALS models, CBD and CBG reduce neuroinflammation and suppress the NF-κB pathway while increasing IL-10 and IL-37 expression [143].

In multiple sclerosis models, a combined treatment of CBD and THC suppresses T cells, reduces the secretion of pro-inflammatory cytokines (IL-17, IFN-γ, and TNF-α), and increases the secretion of anti-inflammatory cytokines (IL-4, IL-10, and TGF-β). These effects are dependent on CB1/CB2 receptors [89].

Most evidence comes from preclinical models, but no animal models of neurodegenerative diseases fully imitate human diseases. Therefore, clinical trials are extremely important in pharmaceutical applications of phytocannabinoids. To systematically illustrate the evidence obtained from these studies, Table 2 summarizes their findings from clinical studies in various neurodegenerative diseases, including the type of cannabinoid, study model, main effects, and mechanisms [90].

Collectively, clinical evidence indicates that phytocannabinoids such as CBD and Δ^9^-THC may provide symptomatic relief in neurodegenerative disorders, including improvements in pain, spasticity, sleep disturbances, and agitation (Table 2). Nevertheless, well-controlled, large-scale clinical trials are required to determine their long-term safety, efficacy, and potential role beyond symptomatic management [90].

## 7. Side Effects

Adverse effects of cannabinoid intake on almost all body systems are well known; their durations and severity depend on many factors, for example, age, sex, concentrations of cannabinoids, and so on [152]. Acidic cannabinoids show mild side effects, while neutral cannabinoids are associated with severe side effects, including psychoactivity [153]. For example, side effects of THC may include various psychoactive effects, tachycardia, dry mouth, red eyes, etc. [154].

It is mostly unclear whether cannabinoids crossing the BBB pose a specific risk to the BBB. In fact, it has been demonstrated in cell and animal models that THC induces BBB damage, which is partly associated with CB1 activation and triggering the oxidative stress response [17].

All adverse events reported in clinical trials are reflected in Table 2.

## 8. Future Directions: Targeting the BBB-ECS Axis

Another key challenge is the strong context dependence of ECS signaling within the NVU. The effects of ECS components on BBB integrity vary depending on the cell type. For example, astrocytic CB1 protects against claudin-5 loss during stress [22], while endothelial CB1 induces oxidative stress and tight junction breakdown [17]. This highlights the importance of studying ECS modulation in a cell-specific and time-dependent manner.

Despite growing interest in the role of phytocannabinoids and the ECS in regulating the BBB, important gaps in knowledge remain. Most mechanistic studies on BBB regulation have focused on CBD and THC. In contrast, acidic phytocannabinoids such as THCA, CBDA, and CBGA have been studied mainly for their pharmacokinetics and general neuroprotective effects [81,92,155]. Multicellular BBB models and *in vivo* studies are necessary to better understand how these acidic compounds affect tight junction organization, transporter function, and neurovascular signaling, particularly given their lower penetration through the BBB compared to neutral counterparts.

Future research should consider combined strategies that target both ECS components and related pathways involved in neuroinflammation and oxidative stress, such as PPAR signaling. Since MAGL and FAAH inhibitors, CB2 activation, and phytocannabinoids like CBD and CBG influence overlapping molecular pathways, rational polypharmacology may provide better protection of BBB integrity and neuronal function than single-target approaches. Moreover, ECS and phytocannabinoids may indirectly influence BBB permeability, NVU function, and neurodegeneration [8]. Changes in microbial metabolites, alteration of circulating cytokine profiles, and modulation of peripheral immune cells may indirectly alleviate BBB stress and enhance barrier integrity and function [156]. This mechanism remains incompletely investigated regarding the endocannabinoid system and phytocannabinoids in neurodegenerative disease models.

The known neurodegenerative disease models require further improvement because the existing models may not allow for the complicated interaction between cellular pathologies and their associated clinical syndromes.

## 9. Conclusions

All the above findings refer to the important roles of the ECS, cannabinoids, BBB, and neurodegeneration in the pathophysiology of neurodegenerative diseases. The various neuroprotective properties of cannabinoids through many cellular and molecular pathways in neurodegenerative diseases have been demonstrated. CBD and THC have been researched much more extensively than many other phytocannabinoids. For example, CBG, CBDV, and acidic cannabinoids have great potential for the treatment of neurodegenerative diseases due to their chemical diversity and ability to interact with various targets. However, they have not been investigated deeply.

Permeability assays, *in vivo* BBB evaluation studies, and formulation strategies to improve delivery are urgently required to define the full therapeutic promise of THCA, CBDA, and other acidic forms of cannabinoids at the BBB.

Outstanding progress has been made in understanding the multifaceted activities of cannabinoids through various mechanisms. However, major gaps still remain, particularly with regard to the imperfect preclinical models and limited clinical studies, with difficulties due to inconsistent methods and small sample sizes. Future investigations of endocannabinoid hydrolytic enzymes should give priority to considering the kinetics of enzyme-catalyzed hydrolysis and search for novel inhibitors or activators; modulating CB1 and CB2 for re-generating balance in neurons and glial cells; reducing degenerative and inflammatory damage; and elucidating the potential mechanisms of cannabinoids for maintaining and increasing BBB integrity.

## Figures and Tables

**Table 1:** Components of the endocannabinoid system at the neurovascular interface and their roles in blood–brain barrier regulation.

Component	Expression at NVU	Main Effects on BBB Structure and Function	Clinical Findings
CB1 receptor	Brain endothelial cells, pericytes, and glial cells [23,25,38]	Modulates tight junction proteins, vascular tone, and oxidative stress [23,38]	CB1 can either preserve or disrupt the BBB; CB1 activation led to downregulation of tight junction proteins [17,26]. Other studies reported that CB1 is upregulated in response to BBB disruption and is responsible for BBB integrity [22,25].
CB2 receptor	Brain endothelial cells, pericytes [23,25], and glial cells [23,47].	Reduces leukocyte adhesion, preserves tight junctions, and limits the release of neurotoxic mediators [21,23].	CB2 activation attenuates BBB leakage and neuroinflammation [21].
GPR55	Brain endothelial cells [27] and glial cells [48].	GPR55 activation leads to transient disruption and reorganization of tight and adherent junction proteins in brain microvascular endothelial cells [27].	Activation of GPR55 reduces BBB integrity and increases BBB permeability *in vivo* [27].
Endocannabinoids (AEA, 2-AG)	Produced by brain endothelial cells, astrocytes, and microglia [15].	Under basal conditions, it may impair endothelial repair, and under injury, it increases claudin-5 and ZO-1 [15,32,39].	Reduced AEA/2-AG levels with elevated degrading enzymes associated with BBB breakdown after traumatic brain injury [42]. AEA/2-AG treatment limits inflammation and preserves BBB integrity in vitro [15,39].
Synthetic enzymes (NAPE-PLD, DAGLα/β)	Brain endothelial and glial cells [22,43,47,49].	increases ECS tone in the brain; DAGLα-derived 2-AG maintains VE-cadherin and ZO-1 in brain endothelial cells [44].	DAGLα inhibition compromises endothelial monolayers and facilitates BBB disruption [44].
Degrading enzymes (FAAH, MAGL)	Brain endothelial, pericytes, and glial cells [22,46,50].	Control 2-AG/AEA and arachidonic acid levels; preserved BBB integrity via anti-oxidative and anti-inflammatory pathways.	MAGL inhibitor protects BBB integrity in an ischemic model [46]; FAAH inhibitor protects the permeability of brain microvascular endothelial cells in vitro [50].
Endocannabinoid-like mediators (PEA, OEA, oleamide)	Brain endothelial and glial cells [29,30,33,34].	PEA and OEA have anti-inflammatory effects on BBB via PPAR signaling; PEA enhances the effects AEA and 2-AG [35]; oleamide inhibits gap-junction communication in glial and microvascular endothelial cells [29,31,32,33,34].	PEA and OEA enhance BBB integrity in vitro and *in vivo* [33,34]. Oleamide increases BBB permeability *in vivo* [31,32].

**Table 2:** Summary of findings from clinical studies in various neurodegenerative diseases.

Disease	Phytocannabinoid	Study Design	Sample Size	Dose	Duration	Phytocannabinoid Effects	Endocannabinoids Modulation	Clinical Findings	Adverse Effects
Multiple Sclerosis	Δ^9^-THC + CBD (Sativex) [144]	Randomized, double-blind, placebo-controlled crossover trial	57 patients	Capsules: 2.5 mg THC + 0.9 mg CBD standardized (escalated to max ~30 mg THC/day)	14 days	May reduce spasticity, alleviates pain, improves sleep	CBD may increase anandamide levels	CB1/CB2-mediated anti-inflammatory modulates glutamatergic neurotransmission	Minor adverse events slightly more frequent in active phase; generally mild toxicity symptoms
Alzheimer’s Disease	Δ^9^-THC/Dronabinol [145]	Randomized, double-blind, placebo-controlled, crossover	12 patients	Oral Dronabinol 2.5 mg twice daily	6 weeks	Increased body weight overall; improved behavior and reduced disturbed behavior more during dronabinol periods	-	CB1/CB2 agonist	Seizure (one patient)
	Nabilone [146]	Randomized, double-blind, placebo-controlled	39 patients	Oral 0.5–1 mg/day	6 weeks	Reduces agitation	-	Improvement in agitation scores compared with placebo	Mild sedation, somnolence
	CBD [147,148]	Randomized, double-blind, placebo-controlled	13 patients	Oral 600 mg/day	4 weeks	Potential neuro-protective and anti-inflammatory effects	Increase anandamide via FAAH inhibition and transport restriction	Antioxidant, anti-inflammatory effects,	Mild gastrointestinal upset, fatigue
Huntington’s Disease	Δ^9^-THC + CBD (Sativex) [149]	Randomized, double-blind, placebo-controlled, crossover pilot clinical trial	24 patients	Oromucosal spray: up to 12 sprays/day	12 weeks	No significant motor, cognitive, behavioral or functional improvement vs. placebo	Not specifically evaluated Increase anandamide via FAAH inhibition and transport restriction	Trial showed safety and tolerability but no significant symptomatic benefit at the prescribed dose	No severe adverse events; well tolerated
Parkinson’s Disease	CBD [150]	Exploratory double-blind trial	21 patients	Oral 150–400 mg/day		Improved non-motor symptoms: sleep disturbances, psychosis; no significant motor improvement	Possible anandamide enhancement, anti-inflammatory	Non CB1/CB2-mediated; antioxidant, anti-inflammatory	Mild somnolence, diarrhea
	CBD [151]	Open-label trial	6 patients	Oral 150–400 mg/day	4 weeks	Reduced psychosis and agitation		Improvement in psychotic symptoms without worsening motor function	No serious adverse events

## Data Availability

No new data were created or analyzed in this study. Data sharing is not applicable to this article.

## Abbreviations

The following abbreviations are used in this manuscript:

Δ^9^-THCA	Δ^9^-Tetrahydrocannabinolic acid
2-AG	2-Arachidonoylglycerol
Aβ	Amyloid-β
AEA	Anandamide
AD	Alzheimer’s disease
ALS	Amyotrophic lateral sclerosis
AQP4	Aquaporine-4
BBB	Blood–brain barrier
BDNF	Brain-derived neurotrophic factor
CB1	Cannabinoid receptor 1
CB2	Cannabinoid receptor 2
CBDA	Cannabidiolic acid
CBDV	Cannabidivarin
CBG	Cannabigerol
CBGA	Cannabigerolic acid
CNS	Central nervous system
CORT	Corticosterone
COX-2	Cyclooxygenase-2
DAGLα	Diacylglycerol lipase α
DOPA	3,4-dihydroxyphenylalanine
EAE	Experimental autoimmune encephalomyelitis
ECS	Endocannabinoid system
FAAH	Fatty acids amide hydrolase
GDNF	Glial cell line-derived neurotrophic factor
GPR55	G-protein-coupled receptor 55
GTS-21	3-(2,4-dimethoxy-benzylidene) anabaseine
HO 1	Heme oxygenase 1
ICAM-1	Intercellular adhesion molecule 1
IFN-γ	Interferon gamma
IL-2	Interleukin-2
IL-6	Interleukin-6
iNOS	Inducible nitric oxide synthase
LDH	Lactate dehydrogenase
LPS	Lipopolysaccharide
M1-like	Macrophages 1-like
M2-like	Macrophages 2-like
MAGL	Monoacylglycerol lipase
MAGLi	Monoacylglycerol lipase inhibitor
MS	Multiple Sclerosis
MPTP	1-methyl-4-phenyl-1,2,3,6-tetrahydropyridine
nAChRs	Nicotinic acetylcholine receptors
NAPE-PLD	N-acyl phosphatidylethanolamine phospholipase D
NF-κB	Nuclear Factor kappa-light-chain-enhancer of activated B cells
NO	Nitric Oxide
NVU	Neurovascular unit
OGD	Oxygen-glucose deprivation
PD	Parkinson’s disease
PEA	N-palmitoyl-ethanolamine
PGE2	Prostaglandin E2
PPARγ	Peroxisome proliferator-activated receptor γ
RhoA	Ras homolog family member A
ROCK	Rho-associated, coiled-coil containing protein kinase
TBI	Traumatic brain injury
TJ	Tight junction
TNF-α	Tumor necrosis factor-α
TRP	Transient receptor potential
TRPA	Transient receptor potential subfamily A
TRPM	Transient receptor potential melastatin
TRPV	Transient receptor potential vanilloid
VCAM-1	Vascular cell adhesion protein 1
VE-cadherin	Vascular endothelial cadherin
VEGF	Vascular endothelial growth factor
ZO-1	Zonula occludens-1
